# Endoscopic endonasal pituitary hemitransposition for resection of suprasellar and retrosellar dermoid cyst

**DOI:** 10.3171/2020.4.FocusVid.19959

**Published:** 2020-04-01

**Authors:** Joao Paulo Almeida, Dennis Tang, Varun R. Kshettry, Raj Sindwani, Pablo F. Recinos

**Affiliations:** 1 Section of Skull Base Surgery, Department of Neurosurgery, Neurological Institute, Cleveland Clinic;; 2 Section of Rhinology, Sinus, and Skull Base Surgery, Department of Otolaryngology, Head and Neck Institute, Cleveland Clinic; and; 3 Minimally Invasive Cranial Base and Pituitary Surgery, Rosa Ella Burkhardt Brain Tumor and Neuro-Oncology Center, Neurological Institute, Cleveland Clinic, Cleveland, Ohio

**Keywords:** endoscopic, pituitary transposition, suprasellar, interpeduncular fossa, skull base surgery, surgical video

## Abstract

This is the case of a 25-year-old woman who had had a previous rupture of a dermoid cyst and now presented with recent MRI scans suggesting further growth of her dermoid cyst. Her lesion was located in the suprasellar space and extended into the interpeduncular fossa and prepontine cistern. Considering the location of the tumor, an endoscopic pituitary hemitransposition was selected for its resection. In this video we present the technical nuances and illustrate the anatomy used for an endoscopic endonasal pituitary hemitransposition for resection of a suprasellar dermoid cyst with extension into the interpeduncular fossa. In this case, a near-total resection was achieved, with no complications and no additional hormonal deficit after surgery.

The video can be found here: https://youtu.be/BHtNf5invUI.

## Transcript

**0:28** This is the case of a 25-year-old woman with a previously diagnosed dermoid cyst. She experienced one episode of seizures and aseptic meningitis 5 years ago due to rupture of the cyst. In clinic she presented with no focal neurological deficits. Recent MRI scans, however, had demonstrated enlargement of the cyst in the last 5 years.

**0:52** Her most recent MRI scan demonstrated the presence of a suprasellar solid cyst lesion suggestive of a dermoid cyst. The lesion was in close contact with the pituitary gland in its posterior and lateral aspect in the left side and also had an extension into the interpeduncular fossa and prepontine cistern. Likely due to the previous rupture of the cyst, a superior portion of it, in close relationship with the right optic tract, had a different radiological characteristic and suggested a more fibrotic component of the tumor. In the CISS sequence, this fibrous part of the tumor is observed as a hypointense segment located in the right aspect of the suprasellar space.

**1:45** Considering the progressive enlargement of the tumor in recent years as well as patient’s preference, it was decided to proceed with an endoscopic endonasal approach for resection of the cyst.

**1:58** The first part of the procedure included harvesting of a right side nasoseptal flap, bilateral posterior ethmoidectomy, posterior septectomy, and a wide sphenoidotomy. The remaining of the procedure is demonstrated in the video as a step-by-step approach.

**2:20** After a wide sphenoidotomy, the posterior part of the sphenoid sinus and the skull base are exposed. In cases such as this, with a presellar sphenoid sinus, the anatomy is not always clear; therefore, an “x-ray vision” of the anatomy is mandatory. Neuronavigation is also useful to confirm the location of sellar and parasellar structures in such cases. Once the location of the sella is identified, drilling is done with a diamond burr and the residual thin bone is removed with a Kerrison rongeur.

**3:00** The next step is to perform a transtuberculum approach, which allows exposure of the suprasellar infrachiasmatic space. With use of a diamond burr, the bone overlying the suprasellar space, in the tuberculum region, is progressively thinned out. A Kerrison rongeur can then be used to remove the last pieces of bone in that space.Bone removal in this region is extended all the way up to the dura fold of the limbus sphenoidale.

**3:41** The next step is to obtain carotid exposure by unroofing the bone overlying the parasellar carotid. Drilling with diamond burr is done to thin out the bone over the carotid. A Kerrison rongeur can be used to remove the residual bone over the carotid. A prominent middle clinoid process is observed and dissected from lateral to medial, away from the clinoid segment of the carotid. Removal of the middle clinoid process allows exposure of the anterior wall of the cavernous sinus as well as further bone removal toward the distal dura ring and optic canal, maximizing the exposure of the suprasellar space. Kerrison rongeurs and high-speed drilling can be used to further expose the carotid and anterior wall of the cavernous sinus. Further drilling is performed to expose the bottom of the sella and to maximize the exposure of the carotid near its genu, near the inferior lateral aspect of the sella.

**4:43** Here we expand the transtuberculum part of the approach to maximize the exposure of the suprasellar space, with bone removal near the medial optic canal on the right side.

**5:10** Further drilling in the inferior aspect of the sella floor is done. Venous bleeding can be encountered while drilling in the skull base in this region, and such venous bleeding can be controlled with use of hemostatic agents and pressure over the bleeding site.

**5:09** Once bone removal is complete, the sella and suprasellar spaces and cavernous sinus are exposed. This anatomical illustration demonstrates the location of the dura opening. Before proceeding with the initial dura cut, a Doppler ultrasound is used to precisely locate the cavernous carotid. A sickle knife is then used to cut the dura overlying the pituitary gland, right medial to the anterior wall of the cavernous sinus. The dura is dissected away from the pituitary gland and then further dura resection is performed. While mobilizing the pituitary gland away from the medial wall of the cavernous sinus, venous bleeding is usually encountered. This can be controlled with use of hemostatic agents and some pressure over the cavernous sinus. The superior intercavernous sinus is coagulated and cut. The dura in the suprasellar space is opened, allowing full exposure of the superior intercavernous sinus, which can then be ligated and cut completely. Venous bleeding from the intercavernous agents and use of a small patty in order to apply pressure over the bleeding point.

**7:20** Once the dura is opened and the arachnoid space is entered, the contents of the tumor are readily visible and then resected. Tumor located in the suprasellar infrachiasmatic space is removed. In order to proceed with removal of the tumor located in the interpeduncular space, the pituitary gland needs to be mobilized laterally and the dura located in the retrosellar space needs to be opened. The intradural pituitary hemitransposition allows exposure of the interpeduncular cistern and further tumor resection in that area. The inferior hypophyseal artery originated from the meningohypophyseal trunk at the posterior genu of the cavernous carotid is encountered and then coagulated and cut in order to allow further mobilization of the pituitary gland. The interpeduncular cistern and its contents are then exposed. The basilar trunk, basilar tip, posterior cerebral arteries, superior cerebellar arteries, and oculomotor nerves are identified. Further mobilization of the pituitary gland and stalk and use of angle endoscopes allow visualization of the upper right corner of the cistern. A fibrous component of the tumor is observed near the perforating branches originated from the basilar tip. Further tumor resection in this area is deemed excessively risky due to the risks of vascular injuries, and therefore further tumor resection is not pursued. This image demonstrates the surgical corridors above and lateral to the pituitary gland used for resection of this tumor.

**9:29** Skull base reconstruction is then done in a multilayer fashion, with DuraGen inlay, an additional dura substitute onlay, supported by fibrillar, followed by positioning of the nasoseptal flap over the bone and dura opening. It is held in place by dura sealants and absorbable nasal packing.

**9:56** Postoperative images demonstrate the near-total resection of the tumor, with a small residual tumor left in the right aspect of the suprasellar space.

**9:48** The patient had an uneventful postoperative course and was discharge home at postoperative day 3. She did not present additional hormonal deficits after surgery.

Long-term follow-up is required for evaluation of endocrine function considering the possibility of new postoperative hypopituitarism after pituitary transposition, as reported in previous studies.
